# Long-term consistent use of a vaginal microbicide gel among HIV-1 sero-discordant couples in a phase III clinical trial (MDP 301) in rural south-west Uganda

**DOI:** 10.1186/1745-6215-14-33

**Published:** 2013-02-01

**Authors:** Andrew Abaasa, Angela Crook, Mitzy Gafos, Zacchaeus Anywaine, Jonathan Levin, Symon Wandiembe, Ananta Nanoo, Andrew Nunn, Sheena McCormack, Richard Hayes, Anatoli Kamali

**Affiliations:** 1 MRC/UVRI Uganda Research Unit on AIDS, Entebbe, Uganda; 2 MRC Clinical Trials Unit, London, UK; 3 Wits Reproductive Health and HIV Institute (WRHI) University of the Witwatersrand, Johannesburg, South Africa; 4 London School of Hygiene and Tropical Medicine, London, UK

**Keywords:** HIV, Vaginal microbicides, Consistent gel use, Adherence, Sero-discordant couples, Phase III trial, Microbicides Development Programme (MDP)

## Abstract

**Background:**

A safe and effective vaginal microbicide could substantially reduce HIV acquisition for women. Consistent gel use is, however, of great importance to ensure continued protection against HIV infection, even with a safe and effective microbicide. We assessed the long-term correlates of consistent gel use in the MDP 301 clinical trial among HIV-negative women in sero-discordant couples in south-west Uganda.

**Methods:**

HIV-negative women living with an HIV-infected partner were enrolled between 2005 and 2008, in a three-arm phase III microbicide trial and randomized to 2% PRO2000, 0.5% PRO2000 or placebo gel arms. Follow-up visits continued up to September 2009. The 2% arm was stopped early due to futility and the 229 women enrolled in this arm were excluded from this analysis. Data were analyzed on 544 women on the 0.5% and placebo arms who completed at least 52 weeks of follow-up, sero-converted or became pregnant before 52 weeks. Consistent gel use was defined as satisfying all of the following three conditions: (i) reported gel use at the last sex act for at least 92% of the 26 scheduled visits or at least 92% of the visits attended if fewer than 26; (ii) at least one used applicator returned for each visit for which gel use was reported at the last sex act; (iii) attended at least 13 visits (unless the woman sero-converted or became pregnant during follow-up). Logistic regression models were fitted to investigate factors associated with consistent gel use.

**Results:**

Of the 544 women, 473 (86.9%) were followed for at least 52 weeks, 29 (5.3%) sero-converted and 42 (7.7%) became pregnant before their week 52 visit. Consistent gel use was reported by 67.8%. Women aged 25 to 34 years and those aged 35 years or older were both more than twice as likely to have reported consistently using gel compared to women aged 17 to 24 years. Living in a household with three or more rooms used for sleeping compared to one room was associated with a twofold increase in consistent gel use.

**Conclusion:**

In rural Uganda younger women and women in houses with less space are likely to require additional support to achieve consistent microbicide gel use.

**Trial registration:**

Protocol Number ISRCTN64716212

## Background

Globally, HIV incidence is still high with about 2.7 million new infections in 2010 [1]. In sub-Saharan Africa, disproportionately more new HIV infections occur among women than men [2]. Consistent condom use could substantially reduce sexually acquired HIV for women, although condom use is dependent on male cooperation [3]. There is an urgent need for additional HIV prevention methods that women can initiate, such as vaginal microbicides. To date, six candidate microbicides have been found to be ineffective in phase IIb or III clinical trials [4-11]. However, the CAPRISA 004 phase IIb trial in mid-2010 provided proof of concept for vaginal microbicides, demonstrating that tenofovir microbicide gel reduced the risk of HIV acquisition for women by 39% [12]. As expected with user-dependent products such as microbicides [13], sub-analyses of the CAPRISA 004 data demonstrated higher levels of effectiveness among women who adhered more strictly to microbicide use. Effectiveness peaked at 54% in women who reported using the microbicide in at least 80% of sex acts [12]. Adherence to microbicides is critical in terms of detecting an effect in clinical trials and reducing the risk of HIV acquisition when an effective microbicide is available [14].

Recent research (largely short-term pilot studies) has found that adherence in microbicide studies was associated with the acceptability of product characteristics [15,16] and instructions for use, an understanding of study concepts, education, partner approval [16] and age [17]. To date, there are no reports regarding adherence to microbicide gels among sero-discordant couples. In this analysis we report on the long-term consistent use of a vaginal microbicide gel among sero-discordant couples in south-west Uganda enrolled as part of the Microbicides Development Programme (MDP) 301 clinical trial. MDP 301 was an international, randomized, double-blind, placebo-controlled parallel-group phase III clinical trial, designed to evaluate the safety and effectiveness of 0.5% and 2% PRO2000 candidate microbicide gels in preventing vaginally acquired HIV-1 infection. Participants were enrolled at 13 clinics, across six research centers in Africa (three in South Africa, one each in Tanzania, Uganda and Zambia). In South Africa, Tanzania and Zambia, women were enrolled without their partners and followed up for a maximum of 52 weeks. In Uganda HIV-negative women in sero-discordant relationships were enrolled and followed up for a maximum of 104 weeks. The trial design and trial results have been reported elsewhere [4,18]. In this analysis we use data from the MDP 301 trial conducted in Masaka, south-west Uganda, to identify predictors of long-term consistent vaginal microbicide gel use among sero-discordant couples.

## Methods

The MDP 301 Masaka clinical trial site enrolled HIV-negative healthy women who were in a known HIV sero-discordant relationship and followed them for a minimum of 52 weeks and a maximum of 104 weeks. Women were enrolled between September 2005 and August 2008 and follow-up visits continued until September 2009. The eligibility criteria are described in Table 1.

**Table 1:** MDP 301 Uganda: Eligibility criteria

**Eligible**	**Ineligible**
Sexually active	Unable or unwilling to provide a reliable method of contact for the field team
16 years old or above	Likely to move permanently out of the study area within the next year
HIV negative at screening	Likely to have sex more than 14 times a week on a regular basis during the course of following up
Willing to undergo regular HIV testing and receive the result before randomization	Using spermicides regularly
Willing to undergo regular speculum examinations and genital infection screens	Pregnant or within six weeks postpartum at enrolment
Willing to have regular urine pregnancy tests	Had a severe clinical or laboratory abnormality
Willing to use study gel as instructed	Requiring referral for assessment of a clinically suspicious cervical lesion
Willing to receive health education about condoms	Had treatment to the cervix, or to the womb through the cervix, within 30 days of enrolment
Willing and able to give informed consent	Had known latex allergy
	Participating, or having participated within 30 days of enrolment, in a clinical trial of an unlicensed product, microbicide, barrier method or any other intervention likely to impact on the outcome of this trial
	Considered unlikely to be able to comply with the protocol

Details of the clinical, laboratory and pharmacy procedures, data management, field activities, counseling package and follow-up schedules are described elsewhere [4,18,19].

After enrolment, follow-up visits were scheduled every four weeks and were conducted at either the research clinic or designated government health centers. Women were initially randomized to one of the three gel groups: 0.5% PRO2000, 2% PRO2000 or placebo. Evaluation of 2% PRO2000 was stopped early on 14 February 2008 on the recommendation of the Independent Data Monitoring Committee on the basis that it was unlikely to show benefit. By February 2008, 229 women had been randomized to the 2% PRO2000 gel arm although only 37 (16.2%) had reached the study end point (either sero-converted or reached 104 weeks of follow-up). This analysis is therefore restricted to the 0.5% PRO2000 and placebo gel arms.

At screening, data were collected on demographic and behavioral characteristics. Behavioral data were also collected at four weekly follow-up visits, including data on gel and condom use at the last sex act. Extended behavioral data were collected at the longer clinical examination visits, which occurred at weeks 4, 12, 24, 40, 52, 64, 76, 88, 100 and 104 after enrolment. Women were asked to bring all used and unused gel applicators to each follow-up visit for reconciliation.

We adapted the predefined ‘consistent’ gel use criteria used for the overall MDP 301 trial for this analysis [4]. As such, consistent gel use was defined as satisfying all of the following three conditions: (i) reported gel use at the last sex act for at least 92% of the 26 scheduled visits or at least 92% of the visits attended if fewer than 26; (ii) at least one used applicator returned for each visit for which gel use was reported at the last sex act; (iii) attended at least 13 (50% of the scheduled visits) of the expected visits (unless the woman sero-converted or became pregnant during follow-up). We included all gel use data up to the point of pregnancy or sero-conversion, although women who sero-converted were invited to continue using gel and this data contributed to the assessment of long-term safety [4].

Although at the longer clinical examination visits, data were collected on gel use at every sex act in the last week or four weeks (or if no sex was reported in the last week), we relied on gel use at the last sex act for the ‘consistent-use’ definition. The rationale for this is that we expect details of the last sex act to be the most accurate and in the overall MDP 301 analysis, on average gel use at the last sex act agreed with gel use at the last 10 sex acts approximately 90% of the time [20].

### Statistical analysis

In this analysis, we included data from women who had completed at least 52 weeks of follow-up or became pregnant or sero-converted prior to the end of follow-up. Women that became pregnant before eight weeks of follow-up were excluded due to having just one observation data point (at the four week visit). The baseline characteristics of trial participants who did not complete 52 weeks of follow-up and those who did were compared using t-tests for continuous variables and chi-square tests for categorical variables. We estimated the proportion of women using gel at a given clinic visit as the number reporting gel use divided by the number of women attending the visit (expressed as a percentage). Associations between consistent gel use and potential demographic, sexual behavior and clinical determinants were examined using odds ratios, with 95% confidence intervals (CIs), by fitting logistic regression models. Only factors for which the association attained statistical significance at the 15% [21] level using a likelihood ratio test (LRT) in an analysis adjusted only for age were considered for the multivariable model. In the multivariable model, factors were removed from the model using a backward elimination algorithm if removing the term did not make the fit of the model significantly worse at the 5% level (on an LRT). To test the robustness of the results, we performed a sensitivity analysis by adding data for participants who were randomized to 2% PRO2000 and had reached at least 52 weeks of follow-up by the time the arm was discontinued.

The MDP 301 definition of consistent gel use was informed by the fact that across all sites, the median reported gel use at last sex act was 92% (94% in the Ugandan dataset). In order to check the fit of the standard MDP 301 definition of consistent gel use to the Uganda dataset, we performed sensitivity analyses based on a median of 94% and arbitrary 90% gel use instead of 92%. Data were analyzed using Stata 10 (StataCorp, College Station, Texas, USA).

### Ethical considerations

The protocol was approved by relevant ethics committees in the UK and in each participating country, including the Uganda Virus Research Institute Science and Ethics committee (Protocol Number ISRCTN64716212) and the Uganda National Council for Science and Technology (UNCST). Authorization was obtained from the national drug regulatory authorities in each participating country, including the Uganda National Drug Authority, and the US Food and Drug Administration. Participants provided written informed consent before being enrolled into the trial.

## Results

**Figure 1:**
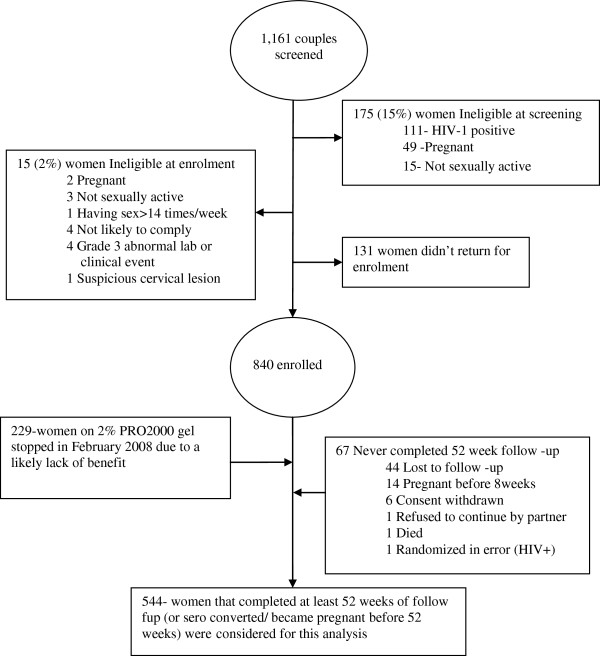
Masaka study profile.

The Ugandan center screened 1,161 women and enrolled 840 (72.4% of those screened); 229 of the 840 (27.3%) were randomized to 2% PRO2000, 307 (36.5%) to 0.5% PRO2000 and 304 (36.2%) to placebo. Figure 1 presents the cohort profile and shows the reasons for ineligibility at screening and enrolment and the number of eligible women who decided not to enroll. As shown in Figure 1, 67 women did not complete 52 weeks of follow-up and were excluded from this analysis. The reasons women did not complete 52 weeks of follow-up were loss to follow-up (44), pregnancy before 8 weeks of follow-up (14), consent withdrawn (6), refusal by partner (1), died (1) and randomized in error (1). Consequently the analysis includes a total of 544 women (64.8% of those enrolled). Of these, 29 (5.3%) sero-converted, 42 (7.7%) became pregnant before 52 weeks of follow-up and 473 (86.9%) reached 52 weeks. Of the 473 women who completed more than 52 weeks of follow-up, 76 (16.1%) had completed 104 weeks of follow-up by the time the study closed.

Table 2 presents the demographic, behavioral and clinical characteristics of the participants. Of the women included in this analysis, over half (60.5%) were less than 35 years old, the majority (85.1%) had attained some formal education and the majority (83.1%) were unemployed. At enrolment 58.3% of women reported the use of a reliable method of family planning and 65.1% reported condom use at last sex. Less than 1% of women reported ever inserting other products intravaginally prior to enrolment. Women reported living in households with a median of five members (IQR 4 to 7) and with a median of two rooms (IQR 1 to 3) used for sleeping. A relatively equal proportion of women reported living in households with 1 (34.4%), 2 (32.5%) or 3 or more (33.1%) rooms used for sleeping.

**Table 2:** Unadjusted and age-adjusted factors for reported consistent gel use

**Characteristic**	***N*(%)**	**Consistent gel users*n*(%)**	**uOR (95% CI)**	**LRT*P*value**	**Age aOR (95% CI)**	**LRT*P*value**
All participants	544 (100)	369 (67.8)				
**Age group**						
17– 24	92 (16.9)	44 (47.8)	1	<0.01	N/A	
25–34	237 (43.6)	172 (72.6)	2.9 (1.8–4.8)			
≥35	215 (39.5)	153 (71.2)	2.7 (1.6–4.5)			
**Religion**						
Christian	472 (86.8)	319 (67.6)	1	0.75	1	0.54
Muslim	72 (13.2)	50 (69.4)	1.1 (0.6–1.9)		1.2 (0.7–2.1)	
**Level of education**						
None	81 (14.9)	54 (66.7)	1	0.81	1	0.52
Some education	463 (85.1)	315 (68.0)	1.1 (0.6–1.8)		1.2 (0.7–2.0)	
**Employment status**						
Employed	92 (16.9)	55 (59.8)	1	0.07	1	0.11
Unemployed	452 (83.1)	314 (69.5)	1.5 (1.0–2.4)		1.5 (0.9–2.4)	
**Family planning use**						
No	227 (41.7)	155 (68.3)	1	0.85	1	0.86
Yes	317 (58.3)	214 (67.5)	0.9 (0.7–1.4)		1.0 (0.7–1.4)	
**Condom use**						
No	190 (34.9)	123 (64.7)	1	0.26	1	0.41
Yes	354 (65.1)	246 (69.5)	1.2 (0.9–1.8)		1.2 (0.8–1.7)	
**Perceived sexual experience using gel**						
Gel made sex less enjoyable	41 (7.5)	26 (63.4)	1	0.48	1	0.27
Gel left sexual pleasure the same	317 (58.3)	209 (65.9)	1.1 (0.6–2.2)		1.2 (0.6–2.4)	
Gel made sex more enjoyable	186 (34.2)	134 (72.0)	1.5 (0.7–3.2)		1.6 (0.8–3.4)	
**Pregnancy status**						
Positive	90 (16.5)	57 (63.3)	1	0.32	1	0.11
Negative	454 (83.5)	312 (68.7)	1.3 (0.8–2.0)		1.5 (0.9–2.5)	
**Rooms used for sleeping in at home**						
One	187 (34.4)	111 (59.4)	1	0.02	1	0.03
Two	177 (32.5)	120 (67.8)	1.4 (0.9–2.2)		1.2 (0.8–1.9)	
Three+	180 (33.1)	138 (76.7)	2.2 (1.4–3.5)		1.9 (1.2–3.1)	
**Trial arm**						
Placebo	275 (50.6)	183 (66.6)	1	0.52	1	0.58
PRO2000 (0.5%)	269 (49.4)	186 (69.1)	1.1 (0.8–1.6)		1.1 (0.8–1.6)	

uOR, unadjusted odds ratio; CI, confidence interval; LRT, likelihood ratio test; Age aOR, age-adjusted odds ratio.

Participants who did not complete at least 52 weeks of follow-up were younger than those who completed at least 52 weeks of follow-up or reached the study end point (mean age of 28.3 vs. 32.7 years, *P* < 0.01). However, there were no other statistically significant differences in terms of religion, educational attainment, employment status, family planning use and condom use at last sex act (data not shown).

**Figure 2:**
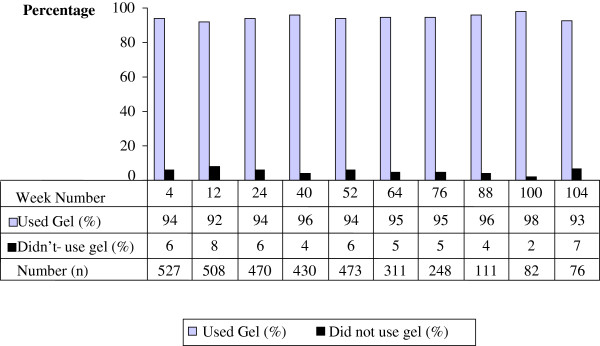
Proportions of women reporting gel use at the last sex act at each clinical visit during follow-up.

The proportion of women reporting to have used gel at their last sex act at clinical examination visits during follow-up ranged from 92.4% to 98.3% (Figure 2). There was no evidence of a marked decline in the point prevalence of gel use over time. Across all visits in the study, the average proportional gel use at last sex act was 92.9% (95% CI: 92.0, 93.9). Based on the predefined criteria, 67.8% (95% CI: 63.7, 71.7) of women reported consistent gel use during the trial. As shown in Table 2, consistent gel use was significantly higher in older participants, at 72.6% among 25 to 34 year olds and 71.2% among women aged 35 years or more, compared to 47.8% among 17 to 24 year olds (*P* < 0.01). Consistent gel use was also significantly higher in women who reported living in households with three or more rooms used for sleeping compared to one room (76.7% vs. 59.4%, *P* < 0.01). Consistent gel use did not differ between the trial arms, and was also not significantly associated with religion, educational attainment, employment status, family planning use, condom use at last sex act, perceived sexual experience of gel use or pregnancy status (Table 2).

In the multivariable model, age group and number of rooms used for sleeping were independently associated with consistent gel use (Table 3). Women aged 25 to 34 years old (aOR = 2.6, 95% CI: 1.6, 4.3) and 35 years or older (aOR = 2.2, 95% CI: 1.3, 3.8) were more than twice as likely to have reported consistently using gel than women aged 17 to 24 years old. Living in a household with three or more rooms used for sleeping was associated with a twofold increase in consistent gel use (aOR = 1.9, 95% CI: 1.2, 3.0). In the sensitivity analyses using the Uganda site median gel use of 94% and arbitrary 90% to define consistent gel users, age group and number of rooms used for sleeping remained the only statistically significant predictors of consistent gel use (data not presented).

**Table 3:** Adjusted factors for reported consistent gel use

**Characteristic**	**Age aOR (95% CI)**	**LRT*P*value**	**aOR (95% CI)**	**LRT*P*value**
All participants				
**Age group**				
17–24	N/A		1	<0.01
25–34			2.6 (1.6–4.3)	
≥35			2.2 (1.3–3.8)	
**Rooms used for sleeping in at home**				
One	**1**	0.03	**1**	0.03
Two	1.2 (0.8–1.9)		1.2 (0.8–1.9)	
Three+	1.9 (1.2–3.1)		1.9 (1.2–3.0)	
**Trial arm**				
Placebo	**1**	0.58	**1**	0.52
PRO2000	1.1 (0.8–1.6)		1.1 (0.8–1.6)	
**Employment status**				
Employed	**1**	0.11	**1**	0.13
Unemployed	1.5 (0.9–2.4)		1.4 (0.8–2.3)	
**Pregnancy status**				
Positive	**1**	0.11	**1**	0.50
Negative	1.5 (0.9–2.5)		1.2 (0.7–1.9)	

Age aOR, age adjusted odds ratio; CI, confidence interval; LRT, likelihood ratio test; aOR, adjusted odds ratio: factors adjusted for age, number of rooms used for sleeping in at home, trial arm allocation, employment and pregnancy status.

## Discussion

We have found that a high proportion of Ugandan women in sero-discordant partnerships used the vaginal gels consistently throughout the trial. The average reported gel use at last sex act (92.9%) was higher than that reported in many other trials, although comparisons are complicated by the use of different measurements. The COL1492 trial, reported approximately 85% of the women using gel in 95% or more vaginal sex acts with clients [10]. In the FHI trials of SAVVY in Ghana and Nigeria, women reported using the gel during approximately 76% and 78% of sex acts respectively, in the last 7 days, based on the participant’s mean gel use across all of their follow-up visits [5,9]. Whereas in the Carraguard trial, in which on average 96% of the women reported using the gel at the last sex act, an applicator stain test to detect vaginal insertion indicated that women only used gel on average during 42% of their sex acts [7]. In the FHI Cellulose Sulphate trial, women reported using gel in more than 80% of sex acts in the previous 7 days at each quarterly visit [22]. Furthermore, in the CONRAD Cellulose Sulphate trial, women reported using the gel in approximately 87% of all sex acts [11] and in the same (CONRAD) trial, adherence was slightly lower in their Uganda site than in the other African sites. The HPTN 035 trial reported women using gel in 81% of their last sex acts, based on self-reported data collected at the quarterly study visits [8]. The CAPRISA 004 trial estimated that on average 72% (median 60%) of self-reported sex acts in the last 30 days were covered by two doses of gel, based on the return of used applicators [12]. Interestingly, the average reported gel use at the last sex act was also higher at the Uganda site than in the overall MDP 301 trial, which was reported at 89% [4]. The reported high average gel use in this analysis may reflect that the women in the MDP 301 trial at Masaka knew that their partner was HIV positive. Known sero-discordance may go some way to explaining differences in adherence, and effectiveness, in recent PrEP trials [22,23]. However, the higher adherence could also have been influenced by the fact that couples were enrolled into the trial together, not just the women. The evidence suggests that generally couples jointly decided on the use of gel and women reported feeling supported by their partners [24].

Few trials have reported on consistent gel use based on a composite measure of self-reported gel use, return of applicators and visit attendance, in the manner used in this analysis. This analysis demonstrated that over two-thirds of women reported consistently using gel for between one and two years. While some trials have observed an increase in gel use over time [12] and others have observed a decrease [9], we did not observe any changes in gel use over time. The main predictors of consistent gel use were age and household space. Few studies to date have reported on predictors of gel adherence [16,17], although in a surrogate study [17] higher age was associated with higher adherence to gel use.

An important finding from this analysis is that consistent gel use was independently associated with the number of rooms in the household used for sleeping. Household space has not been shown to be associated to gel use in any previous studies and was not a predictor of consistent gel use in the overall MDP 301 analysis [25]. Further qualitative research is necessary to understanding the reason for this finding. A possible explanation is that couples living in households with more space enjoy more privacy, thereby making it easier to apply gel before sex. None of the other variables that we evaluated in this analysis were predictive of consistent gel use. Our finding that educational attainment was not significantly associated with consistent gel use is supported by previous findings [16]. It is of particular interest that condom use at the last sex act was not associated with consistent gel use, as gel adherence has been linked to condom use previously [5]. There is substantial evidence that the use of vaginal microbicide gels enhance the sexual pleasure of women and their partners [26-29]. In this analysis, although consistent gel use was slightly higher among women who reported that gel improved sexual pleasure, the impact of the gel on sex was not significantly associated with consistent use.

The strength of this analysis is that we have used a composite measure of consistent gel use. However, the main limitation is that this measure still relies on self-reported sexual behavior and gel use data. Self-reported data are subject to both recall and social desirability bias. In the MDP 301 clinical trial we used a mixed methods and triangulation model to increase the accuracy of adherence and sexual behavior data [30,31]. This model found that women were more likely to under-report sexual activity, condom and gel use in the administered questionnaires used for this analysis, when compared to self-completed diaries and in-depth interviews. Although this model found that most inaccuracies in the self-reported quantitative data were unintentional, there is a chance that consistent gel use has been overestimated in this analysis. Conversely, the fact that the composite measure required women to have attended at least 13 of their expected visits and to have remained in follow-up for at least a year, may have underestimated consistent gel use among women in the trial. Furthermore, underestimation of the consistency of gel use could result from the fact that women who completed fewer visits were more likely to be regarded as inconsistent gel users by the definition; for example, a woman who used gel at the last sex act in 13 out of 15 visits would be regarded as an inconsistent gel user compared to one who completed all the 26 visits and reported using gel at the last sex act in 24 visits. If the women who had attended fewer visits had completed all their scheduled visits, they may well have been considered consistent gel users.

This study suggests that long-term consistent gel use is high among sero-discordant rural couples in south-west Uganda. The fact that adherence was higher in this cohort than previously reported, suggests that factors affecting risk reduction behavior for women in sero-discordant relationships may be distinct from women who are not aware of their partners’ status. The HPTN 052 trial recently demonstrated that early initiation of antiretroviral use by HIV-infected individuals can substantially protect their HIV-uninfected sexual partners from acquiring HIV infection, with a 96% reduction in risk of HIV transmission [32]. Women’s preferences for their own use of vaginal microbicides versus their positive partners’ use of treatment as prevention warrants further research in Uganda. This is the only microbicide trial to report on adherence among sero-discordant couples. However, the evidence regarding the benefit of treatment as prevention may prevent future HIV prevention trials from being able to ethically recruit sero-discordant couples, thereby making it more difficult to assess the use of alternative prevention modalities.

## Conclusion

Our study has demonstrated that rural women in south-west Uganda are willing and able to consistently use vaginal microbicide gels for a year or more. Younger women, who are at highest risk of infection, will require additional support to achieve consistent gel use. Additional qualitative research is necessary to understand the impact of household space on consistent gel use in order to identify support mechanisms for women in households with less space, to enable adherence to gel use. These findings provide optimism regarding the feasibility of the long-term consistent use of vaginal microbicides if an effective microbicide is made available for HIV prevention in the future.

## Abbreviations

*uOR*: *unadjusted odds ratio* ; *aOR*: *adjusted odds ratio* ; *Age* aOR: age-adjusted odds ratio; CI: confidence interval; DFID: Department for International Development; LRT: likelihood ratio test; IQR: Interquartile range; MDP: Microbicides Development Programme; UNCST: Uganda National Council for Science and Technology.

## Competing interests

All authors declare that they have no conflicts of interest.

## Authors’ contributions

Conceived and designed the experiments: AK, SM, RH, AN, MG. Performed the experiments: SW, ZA, AA. Analyzed the data: AA, AC, JL, SW. Wrote the paper: AA, AC, SM, SW, JL, ANA, RH, MG, ZA, AN, AK. All authors read and approved the final manuscript.
